# A systematic review and meta-analysis of acute stroke unit care: What’s beyond the statistical significance?

**DOI:** 10.1186/1471-2288-13-132

**Published:** 2013-10-28

**Authors:** Ying Sun, Dominique Paulus, Maria Eyssen, Johan Maervoet, Omer Saka

**Affiliations:** 1 Deloitte Market Access Strategy & Health Economics, Berkenlaan 8A, 1831 Diegem, Belgium; 2 Belgian Healthcare Knowledge Centre, Boulevard du Jardin Botanique 55, 1000 Brussels, Belgium

## Abstract

**Background:**

The benefits of stroke unit care in terms of reducing death, dependency and institutional care were demonstrated in a 2009 Cochrane review carried out by the Stroke Unit Trialists’ Collaboration.

**Methods:**

As requested by the Belgian health authorities, a systematic review and meta-analysis of the effect of acute stroke units was performed. Clinical trials mentioned in the original Cochrane review were included. In addition, an electronic database search on Medline, Embase, the Cochrane Central Register of Controlled Trials, and Physiotherapy Evidence Database (PEDro) was conducted to identify trials published since 2006. Trials investigating acute stroke units compared to alternative care were eligible for inclusion. Study quality was appraised according to the criteria recommended by Scottish Intercollegiate Guidelines Network (SIGN) and the GRADE system. In the meta-analysis, dichotomous outcomes were estimated by calculating odds ratios (OR) and continuous outcomes were estimated by calculating standardized mean differences. The weight of a study was calculated based on inverse variance.

**Results:**

Evidence from eight trials comparing acute stroke unit and conventional care (general medical ward) were retained for the main synthesis and analysis. The findings from this study were broadly in line with the original Cochrane review: acute stroke units can improve survival and independency, as well as reduce the chance of hospitalization and the length of inpatient stay. The improvement with stroke unit care on mortality was less conclusive and only reached borderline level of significance (OR 0.84, 95% CI 0.70 to 1.00, P = 0.05). This improvement became statistically non-significant (OR 0.87, 95% CI 0.74 to 1.03, P = 0.12) when data from two unpublished trials (Goteborg-Ostra and Svendborg) were added to the analysis. After further also adding two additional trials (Beijing, Stockholm) with very short observation periods (until discharge), the difference between acute stroke units and general medical wards on death remained statistically non-significant (OR 0.86, 95% CI 0.74 to 1.01, P = 0.06). Furthermore, based on figures reported by the clinical trials included in this study, a slightly higher proportion of patients became dependent after receiving care in stroke units than those treated in general medical wards – although the difference was not statistically significant. This result could have an impact on the future demand for healthcare services for individuals that survive a stroke but became dependent on their care-givers.

**Conclusions:**

These findings demonstrate that a well-conducted meta-analysis can produce results that can be of value to policymakers but the choice of inclusion/exclusion criteria and outcomes in this context needs careful consideration. The financing of interventions such as stroke units that increase independency and reduce inpatient stays are worthwhile in a context of an ageing population with increasing care needs. One limitation of this study was the selection of trials published in only four languages: English, French, Dutch and German. This choice was pragmatic in the context of this study, where the objective was to support health authorities in their decision processes.

## Background

Stroke is a major health challenge, particularly for Western healthcare systems. Alongside death it generates serious long-term disability, placing a substantial burden on families and the wider community. Every year 5.5 million people die as a result of having a stroke, accounting for 10% of total deaths worldwide [1]. Even when advanced technology and facilities are available, around 60% of those who suffer a stroke still die or become dependent.

An important intervention in this area of healthcare is the stroke unit, which was introduced during the 1950s [2,3]. This term refers to organized inpatient care for stroke patients, provided by a multidisciplinary team specialized in stroke management [4,5]. The value of stroke units has been extensively investigated in clinical trials and meta-analyses. In particular, the Stroke Unit Trialists’ Collaboration (SUTC) has carried out Cochrane reviews on stroke unit trials since 1997 [4,6,7]. The latest SUTC update concluded that stroke units have a significant impact on patient survival, their likelihood of returning to live at home, and their level of independence [7]. The series of SUTC reviews is widely cited by clinical guidelines and national stroke strategies as the evidence base for their recommendations on stroke unit care [8-10].

In order to advise policymakers on the financing and organization of stroke units, the Belgian Healthcare Knowledge Centre commissioned a three-phase study to evaluate: the efficacy of the services; the criteria used to assess the quality of acute stroke care service provision; and the organization of the stroke units [11]. This study reports the methods and results of the first phase of this endeavor, to answer the question that based on the evidence from clinical trials, how acute stroke unit care can improve patient outcome in terms of mortality and morbidity, compared to alternative care.

## Methods

### Data sources and searches

Trials published prior to 2006 were identified from the 2009 SUTC Cochrane review on stroke units. Only trials in line with the inclusion criteria for this study were retained. An electronic database search was performed in November 2011 on Medline (Ovid), Embase (Ovid), the Cochrane Central Register of Controlled Trials (CENTRAL), and the Physiotherapy Evidence Database (PEDro) to identify trials published after 2006. Online trial registries were also searched (ClinicalTrials.gov and World Health Organization International Clinical Trials Registry Platform) in addition to the indexed databases. Trials were included if they were published in English, French, Dutch or German. Both non-randomized and randomized controlled trials were eligible for inclusion. Search strings were designed by combining medical subject heading (MeSH) terms and text terms (e.g. ‘stroke unit’, ‘clinical trials’) (see Additional file 1). The review protocol is available upon request.

### Study selection and inclusion criteria

The selection of papers was performed independently by two investigators (YS and JM), first on titles and abstracts and then on the full texts, based on the pre-defined inclusion/exclusion criteria. Any disagreement during the study selection process was initially resolved between the two investigators and, when needed, a third reviewer (OS) was involved in the process.

Stroke or ‘stroke-like’ patients who suffered their first symptoms during the seven days prior to hospital admission were qualified for inclusion. The intervention under investigation, stroke unit care, was defined as ‘a geographic location within the hospital designated for stroke or stroke-like patients, staffed by a multidisciplinary team with a special interest and expertise in stroke care’ [4,12]. Any less organized form of care for acute stroke patients was used as a comparator, including care in internal medicine, neurology, cardiology, or geriatric wards. The focus of this study was care for acute stroke patients (i.e. patients admitted to hospital within seven days of stroke onset). Therefore, rehabilitation stroke units (providing only rehabilitation services to stroke patients admitted after the first 7 days) and mixed rehabilitation wards (for stroke and non-stroke patients) did not qualify for either the intervention or the comparator groups. Due to their organizational specificity, mobile stroke teams were also excluded from the meta-analysis. Table 1 below details the PICOS (population, interventions, comparators, outcomes and study designs) elements of this systematic review.

**Table 1:** Inclusion/exclusion criteria in PICOS form

**PICOS element**	**Inclusion criteria**	**Exclusion criteria**
Population	Stroke* or stroke-like patients who had their first symptoms during the past seven days prior to hospital admission, which includes:	Stroke patients who passed the acute phase (first seven days) on symptom onset
	● Patients admitted to hospital for suspected or confirmed recent stroke.	
	● Patients with recent onset of transient ischemic attack (TIA) or other cerebrovascular diseases, as the diagnosis of stroke may be not certain at the admission to the hospital.	
Intervention	Stroke units** that accepted patients in the acute phase of stroke onset, which includes:	● Mixed rehabilitation ward
		● Mobile stroke team
	● Acute stroke unit	● Rehabilitation stroke unit
	● Comprehensive stroke unit	
Comparators	Alternative care for acute stroke	
Outcomes	Studies that reported at least one of the following endpoints:	
	● Number of deaths by the end of the scheduled follow-up	
	● Number of dependent patients by the end of the scheduled follow-up	
	● Number of patients who were institutionalized by the end of the scheduled follow-up	
	● Composite endpoints combined by two of the endpoints mentioned above	
Study design	Randomized or non-randomized controlled trials	

*Clinical definition of stroke: focal neurological deficit due to cerebrovascular disease haemorrhage and subdural haematoma.

**Definition of stroke unit: a geographic location within the hospital designated for stroke and stroke-like patients, staffed by a multidisciplinary team with a special interest and expertise in stroke care.

### Data extraction and quality appraisal

Data extraction was performed by one investigator (YS or JM) and subsequently independently checked by the other investigator (JM or YS) concerning the pre-defined items (e.g. study description, study method, patient characteristics, intervention description, results on primary outcome, results on secondary outcomes and all other outcomes). The two investigators also cross-checked the data extracted from the original manuscripts with those reported by the SUTC Cochrane review on stroke units. In cases where the information identified from the two sources did not match, data from the original manuscript were taken as the primary source for data extraction and analysis. The quality of each included trials was assessed independently by both investigators according to the criteria recommended by Scottish Intercollegiate Guidelines Network (SIGN) [13,14] and the GRADE system [15]. All discrepancies were resolved by arbitration by a third reviewer (OS). Quality of included studies was descriptively synthesized and it was not assessed for the purpose of quantitative analysis.

### Evidence synthesis

The evidence synthesis methodology was in line with the Cochrane review on stroke units. A Peto odds ratio (OR) under a fixed effects assumption was calculated for all dichotomous outcomes. The standardized mean difference was calculated for the comparison of length of stay in a hospital or institution; this approach was taken due to the varied definition of hospital or institutional stay across trials. The fixed effect model was chosen for both dichotomous and continuous outcomes as there was no significant heterogeneity observed on the endpoint with the most complete dataset. Statistical significance was considered as a two-tailed P value smaller than 0.05. A 95% confidence interval was estimated for all comparisons and outcomes at trial level, as well as for pooled evidence. Heterogeneity was assessed by a chi-squared statistic relative to its degrees of freedom and Higgins’ I^2^. All analyses were performed with Review Manager Version 5.1 (the Nordic Cochrane Centre, Copenhagen). Subgroup analysis was performed on different types of acute stroke unit and secondary analysis was performed on a scope of included trials which was comparable to the Cochrane review.

## Results

**Figure 1:**
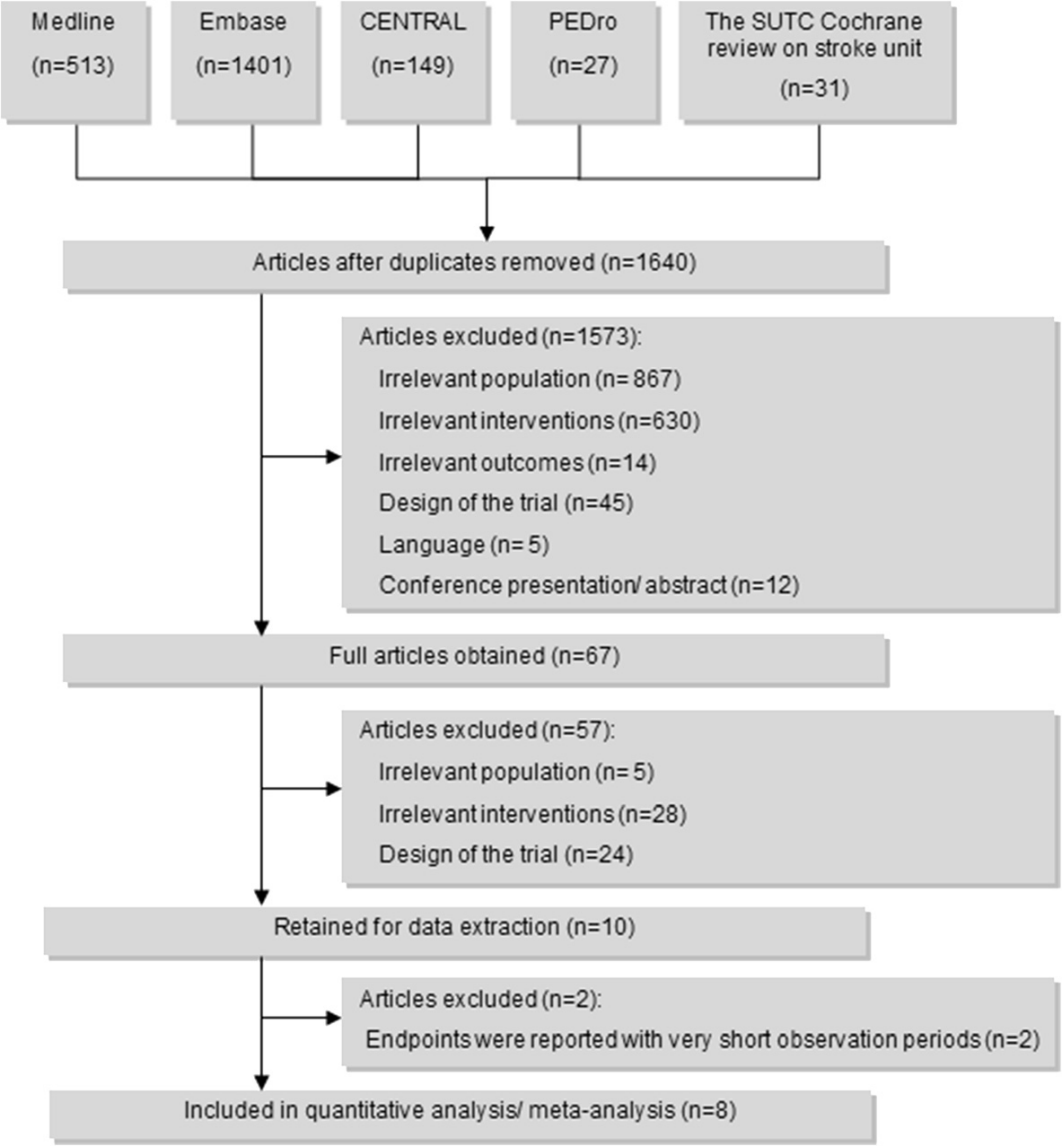
Flow chart of paper identification and selection.

Electronic database search was performed on the 17^th^ of November 2012. Figure 1 summarizes the process of identifying eligible studies. After a first phase in which only titles and abstracts were screened, 67 references were retained for full text screening. After this second round of screening, eight trials were included in the primary analysis. All of these trials were identified from the SUTC Cochrane review of stroke units. One trial [16] published in 2007 met all inclusion criteria but appeared to be a reanalysis of a trial published prior to 2006 [17]. Two trials (Beijing and Stockholm) were excluded from the main analysis due to the very short observation period (from admission until discharge).

### Study characteristics

The characteristics of the eight trials included in the main meta-analysis are summarized in Table 2, together with the two trials with very short observation period (Beijing and Stockholm). Overall there were seven randomized controlled trials and three controlled clinical trials. The quality of the included studies was assessed in accordance with the Scottish Intercollegiate Guidelines Network (SIGN) criteria for controlled clinical trials. Overall, randomization and concealment were poorly addressed across included studies. Risk of bias assessment on individual items can be found in KCE report of stroke unit [11].

**Table 2:** Characteristics of trials included in the meta-analysis

**Trial**	**Design**	**Total number of patients**	**Age**	**Female%**	**Inclusion criteria**	**Stroke unit**	**General medical ward**	**Inpatient stay**	**Follow-up**	Study quality (++, + or -)*
Athens [18,19]	RCT	608	SU: 70.5 GMW: 70.8	Unknown	Acute stroke (onset to admission < 24 h)	Unknown	Unknown	Unknown	1 month 1 year 5 years 6.5 years	-
Akershus [17]	CCT	550	SU: 77 GMW: 76	SU: 47% GMW: 47%	≥ 60 years; acute stroke (onset to admission < 24 h)	Multidisciplinary collaboration + early examination + early mobilization (first hours after admission) + management on fluid, fever, hyperglycemia, hypertension	Good medical treatment without special effort or standardized effort towards this patient group. Patients with hemorrhages were often immobilized for 1 week. No routine of giving antipyretics or parenteral iso-osmolar fluids.	SU: 9.5 days GMW: 7.7 days	7 months	-
Stockholm [20]	CCT	494	SU: 73 GMW: 74	SU: 55% GMW: 63%	Stroke onset within the previous week or TIA onset within last month	Multidisciplinary collaboration + strict criteria for diagnosis and treatment + early active approach to mobilization and rehabilitation	Resources for general patient care in the GMW and SU were not different. Principles of investigation and management of stroke differed, according to routine of consulting physicians	SU: 21 days GMW: 20 days	Till discharge	-
Beijing [21]	RCT	392	62	Unknown	≥ 18 years; stroke	Multidisciplinary collaboration + early mobilization	Unknown	Unknown	Till discharge	-
Edinburgh [22,23]	RCT	311	Unknown	Unknown	≥ 60 years Conscious, established or developing hemiplegia, mean interval from stroke onset to admission: 24 h	Delay in starting physiotherapy treatment in SU: 3 days, in GMW: 3.8 days. No great differences in the use of speech therapy between SU and GMW. More aids or adaptations prescribed in SU to patient at discharge.	Unknown	SU: 55 days GMW: 75 days	60 days 1 year	-
Umea [24]	CCT	293	SU: 72 GMW: 73	SU: 42% GMW: 46%	Acute stroke or TIA ( onset to admission < 7 days)	No facility for intensive care. Multidisciplinary collaboration + early rehabilitation	No standardized program or extra resources for stroke care. Same clinical assessment on admission. Regimes for treatment are uniform.	SU: 21 days GMW: 31 days	1 year	-
Goteborg-Sahlgren [12]	RCT	249	SU: 80 GMW: 80	SU: 66% GMW: 54%	≥ 70 years; Acute stroke (onset to admission < 7 days, 80% in 24 h)	Multidisciplinary collaboration + standard examination on admission + monitoring of body temperature, glucose level, fluid, electrolyte balance + discharge planning	No standardized program or extra resources for the management of stroke patients. CT scan performed in 90% of patients	SU: 28.3 days GMW: 35.8 days	3 weeks 3 months 1 year	++
Trondheim [25]	RCT	220	SU: 72 GMW: 74	SU: 49% GMW: 50%	Acute stroke (onset to admission < 7 days)	Multidisciplinary approach + standard examination (e.g. CT in 24 h of admission) + management on blood pressure, fever, glucose level, fluid, electrolyte balance, cardiac and pulmonary disorders, oxygen	Common treatment for patients with acute stroke in Norwegian hospitals. No standardized program for diagnostic evaluation and treatment	SU: 16 days	52 weeks 5 years 10 years	+
Joinville [26]	RCT	74	SU: 65 GMW: 71	SU: 43% GMW: 41%	Acute stroke (onset to admission < 7 days)	Multidisciplinary collaboration	Routine medical investigation or treatment by neurologists, physiotherapist, occupational therapist were identical to that undertaken at SU	SU: 11 days GMW: 13 days	10 days 1 month 3 months 6 months	-
Perth [27]	RCT	59	SU: 69 GMW: 71	SU: 59% GMW: 47%	Acute stroke (< 7 days duration)	Multidisciplinary collaboration	General physician, medical registrar and resident, ward nurse and allied health staff	SU: 24 days GMW: 27 days	6 months	+

RCT: randomized controlled trial; CCT: controlled clinical trial; SU: stroke unit; GMW: general medical ward; TIA: transient ischemic attack.

*Study quality was checked by the Scottish Intercollegiate Guidelines Network (SIGN) checklist for randomized controlled trials (http://www.sign.ac.uk/methodology/checklists.html).

++: good quality (little bias on randomization, allocation concealment or other biases).

+: acceptable quality (minor bias on randomization, allocation concealment or other biases).

-: low quality (high risk of bias on randomization, allocation concealment or other biases).

The meta-analysis was performed on a total number of patients varying between 824 and 3,246, depending on the settings and endpoints analyzed. The mean age of patients in the included studies ranged from 62 to 80 years; two of the included trials were conducted on patients over 60 years only and one trial on patients over 70 years. The proportion of women in the included trials varied from 39% to 66%. Most trials used ‘acute stroke (or transient ischemic attack)’ as the main inclusion criterion, although the latest admission time varied from 24 hours to one week after symptoms onset. The definition of ‘stroke unit’ also differed between trials, though almost all trials mentioned ‘multidisciplinary collaboration’ as the key feature of a stroke unit. The definition of ‘care in general medical ward’ also varied between studies when it was mentioned in the trial report.

Few trials addressed the extent to which the care in stroke units differed from the care in general medical wards^a^. In some trials, an identical diagnosis and treatment strategy was applied in both settings. The difference between the intervention (stroke unit care) and the comparator (general medical ward care) was wide-ranging, with the most structured form of stroke unit being comprised of multidisciplinary collaboration, early examination, early mobilization (first hour after admission) and care protocol [17]. The least organized form of stroke unit was characterized by multidisciplinary collaboration only [26,27].

### Effects of acute stroke unit care

**Figure 2:**
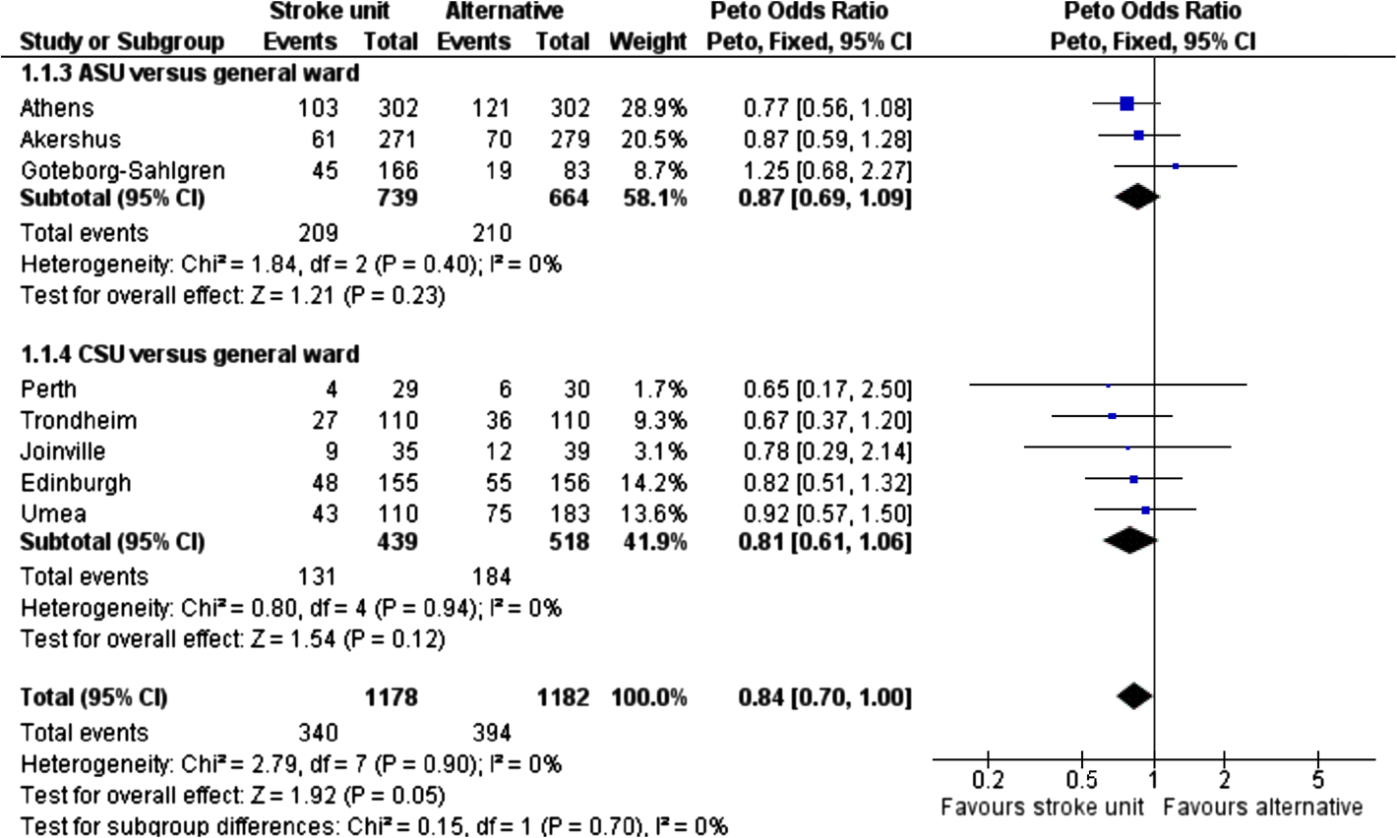
Meta-analysis result of stroke unit versus general medical ward: death by the end of scheduled follow up.

The benefit of acute stroke unit care was of borderline statistical significance on mortality (OR 0.84, 0.70-1.00, P = 0.05, I^2^ = 0%, see Figure 2). Two subgroups were introduced into the meta-analysis: acute stroke unit (ASU) and comprehensive stroke unit (CSU). Acute stroke units admit patients in the acute phase but discharge early (usually within seven days). Comprehensive stroke units admit patients in the acute phase but also provide rehabilitation for at least one week if necessary. No significant difference was observed in the efficacy of stroke unit care across these two subgroups (P for subgroup difference: 0.70). The odds ratio of death for those treated in stroke units was estimated at 0.84 (0.70 - 1.00) compared to general medical ward care. Stroke unit care was significantly more efficacious than general medical ward care for the following endpoints: independency (OR 1.23, 95% CI 1.04 to 1.45, I^2^ = 42%, P = 0.01) death or institutional care (OR 0.70, 0.60 to 0.83, I^2^ = 0%, P < 0.0001), institutional care (OR 0.61, 95% CI 0.47 to 0.79, I^2^ = 39%, P = 0.0002), death or dependency (OR 0.81, 95% CI 0.69 to 0.96, I^2^ = 42%, P = 0.01), and length of stay (days) in a hospital or institution or both (standardized mean difference −1.79 days, 95% CI −2.53 to −1.04, I^2^ = 78%, P < 0.00001). No significant improvement in dependency was observed post stroke unit care (OR 0.92, 95% CI 0.74 to 1.13, I^2^ = 46%, P = 0.42).

### Divergence between the results of this study and the Cochrane review on organized inpatient care for stroke (the 2009 SUTC study)

Apart from the results on mortality, the findings of this meta-analysis were in line with the 2009 SUTC Cochrane review on stroke units. In the Cochrane review, a subgroup analysis of comprehensive stroke wards (a type of stroke unit comparable to the acute stroke units included in this study) was related to a significant reduction of death (OR 0.85, 0.72–0.99, P = 0.03), death or institutional care (OR 0.80, 0.70-0.92, P = 0.0015) and death or dependency (OR 0.83, 0.71-0.97, P = 0.017) [7]. No meta-analysis was performed on dependency or independency as individual endpoints in the Cochrane review.

#### Mortality: from significance to non-significance

Given the varied inclusion/exclusion criteria of the two reviews, some difference in the results was expected, as well as in the extent of variation. Both published and unpublished data were included in the Cochrane review, whereas the latter were excluded in this study. In addition, trials with very short observation periods (until discharge) were also excluded from the main analysis in this study. All these differences are summarized in Table 3, together with their impact on the odds ratio of the endpoint ‘death by the end of scheduled follow up’. As presented in Table 3, inclusion of trials with unpublished data only (Goteborg-Ostra and Svendborg) drove the P value from 0.05 to 0.12 (non-significant), due to higher mortality in stroke units compared to general medical wards in these two trials. On the other hand, inclusion of trials with very short observation (until discharge; Beijing and Stockholm) tended to increase the statistical significance (P = 0.06), albeit still not reaching the 0.05 significance threshold. The increase of statistical significance caused by adding the two trials with very short observation could be explained in terms of the increased statistical power that comes with a bigger sample size.

**Table 3:** Comparison between the Cochrane review and this study in terms of scope and impact on P value in mortality

	**OR (95% CI)**	**P value**
**Present study - Primary analysis**		
Eight included trials (n = 8; Athens corrected*, Akershus, Goteborg-	0.84 (0.70 – 1.00)	P = 0.05
Sahlgren, Perth, Trondheim, Joinville, Edinburgh, Umea)		
**Present study - Secondary analysis**		
✓ Inclusion of two unpublished trials	0.87 (0.74 – 1.03)	P = 0.12
(n = 10 ; Same as above plus Goteborg-Ostra, Svendborg)		
✓ Inclusion of trials with very short-term observation	0.86 (0.74 – 1.01)	P = 0.06
(n = 12 ; Same as above plus Beijing, Stockholm)		
(i.e. All trials included in the Cochrane review, Athens corrected*)		
**The Cochrane review 2009**		
✓ Cochrane review – Analysis 2.1 subtotal	0.85 (0.72 – 0.99)	P = 0.03
(n = 12; all trials mentioned above, Athens uncorrected)		

* Number of deaths in the control arm was 121/302 instead of the 127/302 mentioned in the Cochrane Review 2009.

One interesting finding came from an evaluation of the Athens trial [18,19] as it appeared there was a difference between the number of deaths in the control arm mentioned in the original manuscript (121/302 deaths in the control arm) and those reported in the Cochrane review (127/302 deaths in the control arm). In correspondence with the author of the Cochrane review, this was explained as an error which will be corrected in the next update of the Cochrane review. This error did not only affect the interpretation of the results for this individual trial (OR 0.77; 95% CI 0.56 to 1.08 versus OR 0.71; 95% CI 0.51 to 0.99) but, given the large sample size of the Athens trial, also changed the significance of the benefit of acute stroke unit care compared with general medical ward care regarding the mortality measure in the Cochrane review. After correction of the error, the effect of acute stroke unit care on mortality in the Cochrane review becomes statistically non-significant (P = 0.06) instead of significant (P = 0.03) (Table 3).

#### Why was dependency not reduced with acute stroke units?

The results of the present study suggest that acute stroke unit care was not superior to general medical ward care in terms of reducing dependency (OR 0.92, 0.74 to 1.13, P = 0.42). However, this finding should be interpreted with caution from both a clinical and a policy perspective.

**Figure 3:**
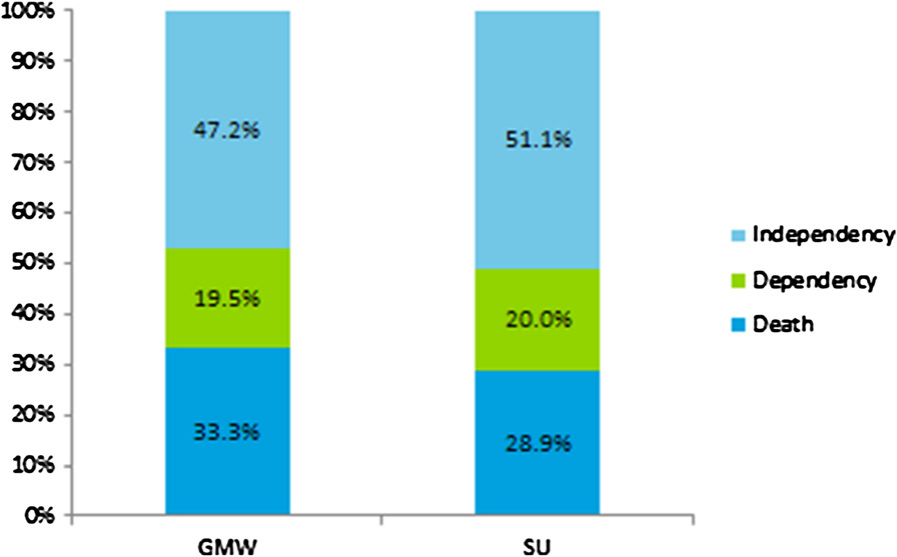
Proportion of deaths, dependent and independent cases reported by clinical trials included in this study.

From a clinical perspective, the outcome ‘dependency’ is different from ‘death’ or ‘independency’, which are endpoints implying a definitive clinical deterioration or improvement. In this study, the eight trials included in the main meta-analysis reported data on both death and dependency; these showed 33.3% of the patients treated in general medical wards and 28.9% of the patients treated in stroke units were dead within one year after stroke onset (Figure 3). Meanwhile, 19.5% and 20.0% of the patients treated in general medical wards and stroke units became dependent (i.e. stroke unit care was associated a slight increase in the risk of dependency). To complete the picture, respectively 47.2% and 51.1% of the patients treated in general medical wards and stroke units became independent (i.e. stroke unit care was also associated with a higher chance of independency). The figures thus imply that stroke unit care saved some patients from death, some of whom became independent but some did not. This explains why there was an increase in the number of dependent cases in the stroke units included in the clinical trials. As such, the increase in dependent cases within stroke units should be taken as a clinical *improvement* rather than a deterioration.

While accepting it as a benefit, an increased overall risk of dependency implies that stroke unit care may result in higher demand for healthcare service for dependent individuals, compared with general medical ward care, precisely as a result of an increased survival rate.

**Figure 4:**
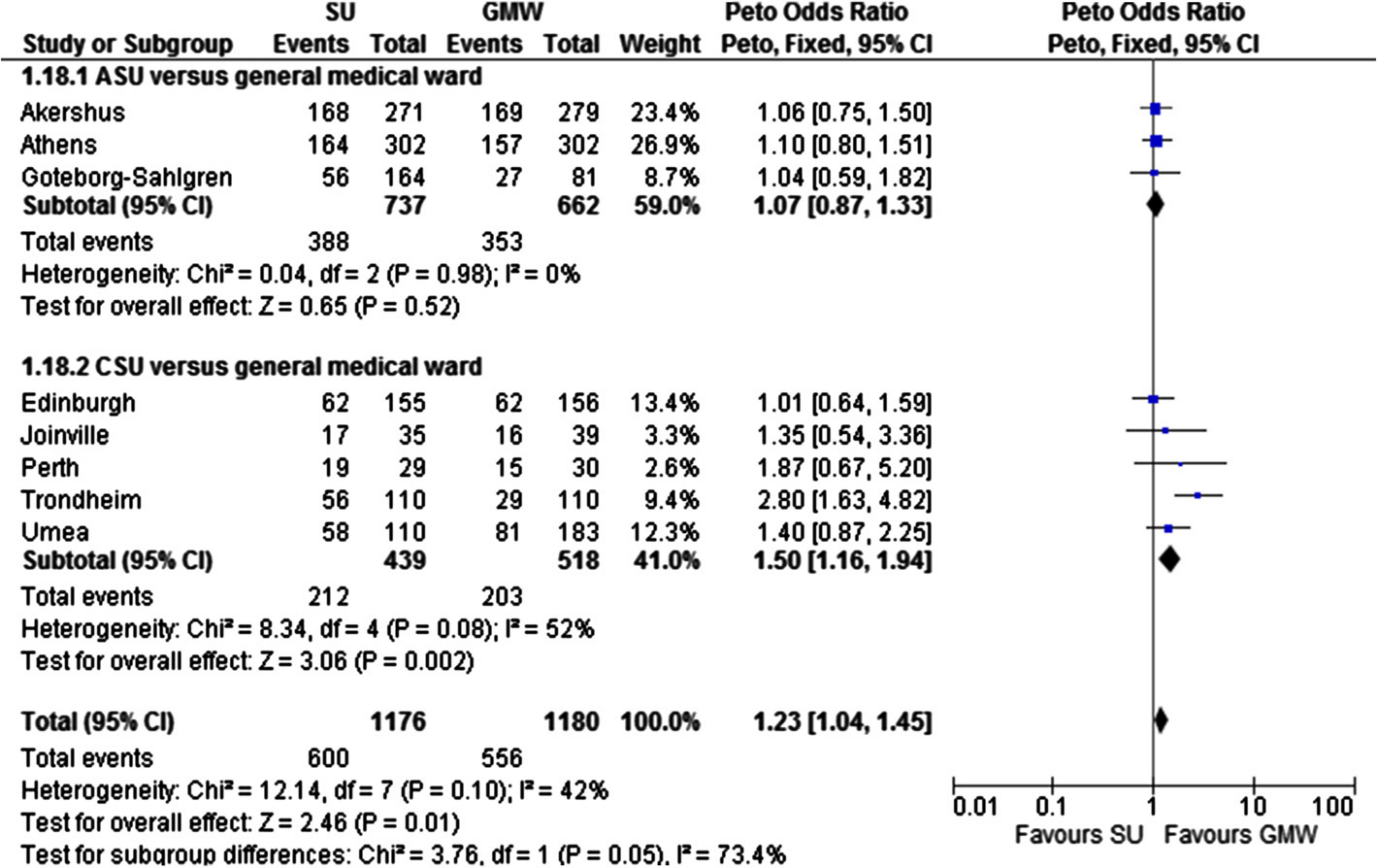
Meta-analysis result of stroke unit versus general medical ward: independency by the end of scheduled follow up.

Readers should note that the composite endpoint ‘death or dependency’ not surprisingly yielded the same results as ‘independency’, due to the fact that these three outcomes are mutually exclusive (‘death or dependency’ is equal to ‘not independency’). In Figure 4, ‘independency’ is used as an endpoint to compare stroke unit and general medical care, resulting in an identical P value (P = 0.01), inversed odds ratio (OR = 1.23 = 1/0.81) and 95% confidence interval (1.04 = 1/0.96 to 1.45 = 1/0.69) as when using the endpoint ‘death or dependency’.

## Discussion

The inclusion/exclusion criteria of a systematic review should be governed by the context of the study and its research question. However, the choice of criteria can sometimes be difficult due to its potential impact on the study’s results and conclusions. A broad set of inclusion criteria may result in the inclusion of a large number of heterogeneous trials of varying quality, but may enhance the statistical power to test significance. On the other hand, tight inclusion criteria can ensure a good level of homogeneity and better quality of included evidence, but may hamper the statistical power especially if there are very few trials published in the field of research. Similar trade-offs occur when deciding whether to include unpublished trials/data, which may alleviate publication bias but may also lower data quality and/or introduce other types of bias. This point is demonstrated by this study where, for the same outcome (mortality), the results on acute stroke unit care differed from the results of the Cochrane review. Most of the findings from this study were in line with the Cochrane review: acute stroke units can improve survival and independency, as well as reduce the chance of hospitalization and length of inpatient stay. However, the benefit of acute stroke units on mortality appears less conclusive: the statistical significance appeared to be very sensitive to the inclusion/exclusion criteria (e.g. inclusion of unpublished trials) and to an error in the Cochrane analysis relating to the data from a large-scale trial.

This study also found no significant improvement in the risk of dependency when comparing stroke unit care with general medical ward care (236/1178 versus 230/1182, P = 0.42). This may potentially elevate the demand for healthcare services that will be required by patients who survive but continue living in a state of dependency. It should be noted that stroke severity at the baseline could have an impact on the outcome of stroke care: those with severe stroke may be more likely to survive but with a degree of disability; those with moderate stroke may be more likely to survive and regain independence; and those with mild stroke may be likely to regain independence. Thus when the benefit of acute stroke unit care on survival is so accentuated that it masks its benefit on independency, the increased number of dependent cases could result in an even higher demand for healthcare resources for dependent patients. Apart from the implication for health policymaking, some experts involved in this study underlined the fact that in some cases dependency could be worse than death when taking into account its impact on patients’ quality of life, which makes it a challenge to interpret the slightly increased number of dependent patients in stroke units observed in clinical trials.

In Belgium, stroke units exist but currently there is no formal accreditation process to assess their compliance with a set of official standards. As a consequence, there is a large variability in the structure, process and (probably) the quality of care provided to stroke patients. Some hospitals have not yet established stroke units. This can be for various reasons, such as lack of awareness and knowledge of the benefits of stroke units, financial barriers due to the additional equipment and personnel needed, lack of protocols, insufficient staffing, and lack of collaboration between medical departments involved in acute stroke care (e.g. emergency, neurology, radiology, etc.).

As the first of a series of studies on organized inpatient care for stroke, the evidence on the efficacy of acute stroke units as demonstrated in this study suggests that there would be an overall improvement in care quality if all eligible patients in Belgium were to have access to care in acute stroke units.

The results of this study have been presented to the Belgian stakeholders, including representatives of the Federal Government. Different scenarios for the implementation of stroke units in Belgium have been suggested: one stroke unit in each hospital; highly specialized units in a limited number of hospitals; a combination of these two solutions, i.e. highly specialized care in some specific centers followed by subacute care in local hospitals; and thrombolysis in every hospital, with stroke units in specialized centers. After weighting the pros and cons of each alternative, the authors’ policy recommendation has focused on the third option (highly specialized centers followed by care in local hospitals). This solution would combine acute specialized care and accessible high quality subacute care in the most efficient way. The recommendations also advised the development of quality indicators to monitor the quality of stroke care. Last but not least, an emphasis has been put on the organization of a seamless transfer from the hospital to primary care services and the patient’s normal daily living environment (own home or residential care). The final decision of the federal Government is awaited.

### Limitations

One limitation of this study was the selection of trials published in only four languages: English, French, Dutch and German. This choice was pragmatic in the context of this study, where the objective was to support health authorities in their decision processes. A future analysis of this type of restriction on publication language could be carried out in order to evaluate the impact it had on the conclusions of this study.

## Conclusions

The findings from this study are broadly in line with the Cochrane review: acute stroke unit care can improve survival and independency, as well as reduce the chance of hospitalization and the length of inpatient stay. However, the effect on mortality remains less conclusive. These results demonstrate that a well-conducted meta-analysis can produce results that can be of value to policymakers but the choice of inclusion/exclusion criteria and outcomes in this context needs careful consideration. The financing of interventions such as stroke units that increase independency and reduce inpatient stays are worthwhile in a context of an ageing population with increasing care needs.

## Endnote

^a^The Cochrane review on organized inpatient care for stroke included an interview with individual trialists that used a standard set of criteria. Thus more descriptive information is available in that review than in other published papers.

## Abbreviations

ASU: Acute stroke unit; CCT: Controlled clinical trial; CENTRAL: Cochrane Central Register of Controlled Trials; CI: Confidence interval; CSU: Comprehensive stroke unit; GMW: General medical ward; MeSH: Medical subject heading; OR: Odds ratio; PEDro: Physiotherapy evidence database; RCT: Randomized controlled trial; RevMan: Review Manager; SIGN: Scottish Intercollegiate Guidelines Network; SU: Stroke unit; SUTC: Stroke Unit Trialists’ Collaboration; TIA: Transient ischemic attack.

## Competing interests

The authors declare that they have no competing interests.

## Authors’ contributions

YS and DP participated in the design of the study. YS and JM performed paper selection and data extraction. YS performed the statistical analysis and drafted the manuscript. ME and OS participated in coordination and execution of the KCE Stroke Unit project. All authors critically reviewed and made important intellectual contributions to this manuscript. All authors read and approved the final manuscript.

## Pre-publication history

The pre-publication history for this paper can be accessed here:

http://www.biomedcentral.com/1471-2288/13/132/prepub

## Supplementary Material

Additional file 1 Literature search strings used in medline, embase and cochrane central register of controlled trials. Click here for file
