# Supplementary material for: A systematic review and meta-analysis of acute stroke unit care: What’s beyond the statistical significance?
Source: BMC Med Res Methodol. 2013 Oct 28;13:132. doi: 10.1186/1471-2288-13-132 (PMC4231396; doi:10.1186/1471-2288-13-132)
Supplement: Additional file 1 — Literature search strings used in medline, embase and cochrane central register of controlled trials. [file 1471-2288-13-132-S1.docx]

# Additional file 1: literature search strategy

Ovid MEDLINE(R) In-Process & Other Non-Indexed Citations and Ovid MEDLINE(R) <1948 to Present> (Date of search: 17/11/2011)

1 exp stroke/ (68881)

2 (stroke* or apoplexy or cerebrovascular accident* or brain vascular

accident* or cvas or cva or cerebral vascular accident*).tw. (129247)

3 Ischemic attack, transient/ (16606)

4 (transient brainstem isch?emia* or transient cerebral isch?emia* or

transient isch?emic attack* or tia or tias).tw. (11188)

5 exp cerebrovascular disorder/ (244218)

6 3 or 4 (22957)

7 2 and 6 (8847)

8 2 and 5 (72631)

9 1 or 7 or 8 (97950)

10 exp hospital units/ (67491)

11 *hospital, special/ (6058)

12 hospital departments/ (13779)

13 intensive care/ (13148)

14 ((inpatient adj3 care) or unit* or ward*).tw. (575494)

15 or/10-14 (632058)

16 9 and 15 (5527)

17 ((stroke adj3 unit*) or (stroke adj3 ward*) or (stroke adj3 team*) or

inpatient stroke care or inpatient stroke management).tw. (2076)

18 16 or 17 (5822)

19 Randomized Controlled Trials as Topic/ (78000)

20 randomized controlled trial/ (322382)

21 Random Allocation/ (73633)

22 Double Blind Method/ (113969)

23 Single Blind Method/ (15763)

24 clinical trial/ (470464)

25 clinical trial, phase i.pt. (11888)

26 clinical trial, phase ii.pt. (18798)

27 clinical trial, phase iii.pt. (6701)

28 clinical trial, phase iv.pt. (670)

29 controlled clinical trial.pt. (84016)

30 randomized controlled trial.pt. (322382)

31 multicenter study.pt. (140010)

32 clinical trial.pt. (470464)

33 exp Clinical Trials as topic/ (252284)

34 or/19-33 (897551)

35 (clinical adj trial$).tw. (174044)

36 ((singl$ or doubl$ or treb$ or tripl$) adj (blind$3 or mask$3)).tw.

(114686)

37 PLACEBOS/ (30721)

38 placebo$.tw. (139063)

39 randomly allocated.tw. (13788)

40 (allocated adj2 random$).tw. (16128)

41 or/35-40 (356657)

42 34 or 41 (1012785)

43 case report.tw. (176257)

44 letter/ (749641)

45 historical article/ (284364)

46 or/43-45 (1199942)

47 42 not 46 (985764)

48 18 and 47 (1202)

49 "outcome and process assessment (health care)"/ or "outcome

assessment (health care)"/ or "process assessment (health care)"/

(62912)

50 program evaluation/ (39686)

51 quality indicators, health care/ (7925)

52 clinical indicator$.tw. (1786)

53 performance indicator$.tw. (1418)

54 performance outcome$.tw. (450)

55 quality indicator$.tw. (2954)

56 performance standard$.tw. (917)

57 quality measure*.tw. (2693)

58 outcome measure*.tw. (116356)

59 exp Quality Assurance, Health Care/ (225419)

60 benchmarking/ (8749)

61 or/49-60 (424663)

62 18 and 61 (1253)

63 62 not 46 (1226)

64 limit 48 to (yr="2006 -Current" and (dutch or english or french or

german)) (513)

65 limit 63 to (yr="2000 -Current" and (dutch or english or french or

german)) (989)

Embase <1980 to 2011 Week 45> (Date of search: 17/11/2011)

1 Stroke/ (107321)

2 (stroke* or apoplexy or cerebrovascular accident* or brain vascular

accident* or cvas or cva or cerebral vascular accident*).tw. (160509)

3 transient ischemic attack/ (19268)

4 (transient brainstem isch?emia* or transient cerebral isch?emia* or

transient isch?emic attack* or tia or tias).tw. (14400)

5 exp cerebrovascular disease/ (345400)

6 3 or 4 (25111)

7 2 and 6 (11823)

8 2 and 5 (117842)

9 Stroke patient/ (5254)

10 1 or 7 or 8 or 9 (148875)

11 ((inpatient adj3 care) or unit* or ward*).tw. (749442)

12 10 and 11 (10374)

13 Stroke unit/ (1086)

14 ((stroke adj3 unit*) or (stroke adj3 ward*) or (stroke adj3 team*) or

inpatient stroke care or inpatient stroke management).tw. (3306)

15 or/12-14 (10800)

16 Clinical trial/ (820810)

17 Randomized controlled trial/ (292216)

18 Randomization/ (54949)

19 Single blind procedure/ (14402)

20 Double blind procedure/ (101570)

21 Crossover procedure/ (31137)

22 Placebo/ (187119)

23 Randomi?ed controlled trial$.tw. (66039)

24 Rct.tw. (7970)

25 Random allocation.tw. (1064)

26 Randomly allocated.tw. (15769)

27 Allocated randomly.tw. (1715)

28 (allocated adj2 random).tw. (688)

29 Single blind$.tw. (11198)

30 Double blind$.tw. (118974)

31 ((treble or triple) adj blind$).tw. (249)

32 Placebo$.tw. (161172)

33 Prospective study/ (176077)

34 or/16-33 (1154917)

35 Case study/ (13740)

36 Case report.tw. (209839)

37 Abstract report/ or letter/ (798669)

38 or/35-37 (1018157)

39 34 not 38 (1121429)

40 15 and 39 (2586)

41 limit 40 to ((dutch or english or french or german) and yr="2000 -

Current") (2171)

42 "evaluation and follow up"/ (1810)

43 clinical assessment/ (41868)

44 clinical evaluation/ (25282)

45 course evaluation/ (1143)

46 outcome assessment/ (142615)

47 health care quality/ (151801)

48 clinical indicator/ (722)

49 performance measurement system/ (1825)

50 professional standard/ (20472)

51 quality of nursing care/ (94)

52 quality circle/ (41)

53 total quality management/ (14607)

54 quality control/ (95302)

55 ((Performance or clinical or Quality) adj (indicator* or criteria or

stand* or measure*)).tw. (39412)

56 limit 40 to ((dutch or english or french or german) and yr="2006 -

Current") (1401)

57 or/42-55 (490162)

58 57 not 38 (466159)

59 58 and 15 (1163)

60 limit 59 to ((dutch or english or french or german) and yr="2000 -

Current") (1057)

Cochrane Central Register of Controlled Trials (Date of search:17/11/2011)

ID Search Hits

#1 MeSH descriptor Stroke explode all trees 3785

#2 (stroke* or apoplexy or cerebrovascular accident* or brain vascular accident* or cvas or cva or cerebral vascular accident*):ti,ab 15329

#3 MeSH descriptor Ischemic Attack, Transient, this term only 458

#4 (transient brainstem isch?emia* or transient cerebral isch?emia* or transient isch?emic attack* or tia or tias):ti,ab 523

#5 MeSH descriptor Cerebrovascular Disorders explode all trees 7541

#6 (#3 OR #4) 810

#7 (#2 AND #6) 573

#8 (#2 AND #5) 4303

#9 (#1 OR #7 OR #8) 5167

#10 MeSH descriptor Hospital Units explode all trees 2391

#11 MeSH descriptor Hospitals, Special, this term only 40

#12 MeSH descriptor Hospital Departments, this term only 49

#13 MeSH descriptor Intensive Care, this term only 723

#14 ((inpatient adj3 care) or unit* or ward*):ti,ab 25548

#15 (#10 OR #11 OR #12 OR #13 OR #14) 26859

#16 (#9 AND #15) 425

#17 ((stroke adj3 unit*) or (stroke adj3 ward*) or (stroke adj3 team*) or inpatient stroke care or inpatient stroke management):ti,ab 68

#18 (#16 OR #17) 470

#19 (#16 OR #17), clinical trials 437

#20 (#16 OR #17), from 2006 to 2011 149
